# Methanolic Extract of *Boswellia serrata* Gum Protects the Nigral Dopaminergic Neurons from Rotenone-Induced Neurotoxicity

**DOI:** 10.1007/s12035-022-02943-y

**Published:** 2022-07-08

**Authors:** Sina Shadfar, Shristi Khanal, Ganesh Bohara, Geumjin Kim, Saeed Sadigh-Eteghad, Saeid Ghavami, Hyukjae Choi, Dong-Young Choi

**Affiliations:** 1 Centre for Motor Neuron Disease Research, Macquarie Medical School, Faculty of Medicine, Health and Human Sciences, Macquarie University, Sydney, 2121 NSW Australia; 2 College of Pharmacy, Yeungnam University, 280 Daehak Avenue, Gyeongsan, Gyeongbuk 38541 Republic of Korea; 3 Neurosciences Research Center, Tabriz University of Medical Sciences, Tabriz, Iran; 4 Department of Human Anatomy and Cell Science, University of Manitoba College of Medicine, Winnipeg, MB R3E 0V9 Canada; 5 Research Institutes of Oncology and Hematology, Cancer Care Manitoba-University of Manitoba, Winnipeg, MB R3E 0V9 Canada; 6 Autophagy Research Center, Shiraz University of Medical Sciences, Shiraz, 7134845794 Iran; 7 Faculty of Medicine, Katowice School of Technology, 40-555 Katowice, Poland

**Keywords:** Parkinson’s disease, Boswellia serrata, Rotenone, Neurodegenerative disease, Mice, Locomotor

## Abstract

*Boswellia serrata* gum is a natural product that showed beneficial effects on neurodegenerative diseases in recent studies. In this study, we investigated the effects of *Boswellia serrata* resin on rotenone-induced dopaminergic neurotoxicity. Firstly, we attempted to see if the resin can induce AMP-activated protein kinase (AMPK) signaling pathway which has been known to have broad neuroprotective effects. Boswellia increased AMPK phosphorylation and reduced phosphorylation of mammalian target of rapamycin (p-mTOR) and α-synuclein (p-α-synuclein) in the striatum while increased the expression level of Beclin1, a marker for autophagy and brain-derived neurotrophic factor. Next, we examined the neuroprotective effects of the Boswellia extract in the rotenone-injected mice. The results showed that Boswellia evidently attenuated the loss of the nigrostriatal dopaminergic neurons and microglial activation caused by rotenone. Moreover, Boswellia ameliorated rotenone-induced decrease in the striatal dopamine and impairment in motor function. Accumulation of α-synuclein meditated by rotenone was significantly ameliorated by Boswellia. Also, we showed that β-boswellic acid, the active constituents of *Boswellia serrata *gum, induced AMPK phosphorylation and attenuated α-synuclein phosphorylation in SHSY5 cells. These results suggest that Boswellia protected the dopaminergic neurons from rotenone neurotoxicity via activation of the AMPK pathway which might be associated with attenuation of α-synuclein aggregation and neuroinflammation. Further investigations are warranted to identify specific molecules in Boswellia which are responsible for the neuroprotection.

## Introduction

Parkinson’s disease (PD) is the second most prevalent form of neurodegenerative disorder after Alzheimer’s disease. The disease causes selective loss of dopaminergic neurons in the substantia nigra pars compacta leading to a significant reduction of dopamine (DA) in the striatum [1]. Despite the long history of the disease, the exact etiology of PD remains unclear and it has been posited that multiple factors may contribute to PD incidence and progress [2]. Currently, there is no approved treatment for the prevention or attenuation of the progressive neurodegeneration in parkinsonian patients.

A remarkable and consistent biomarker for PD is the Lewy body which is a cytoplasmic inclusion caused by the aggregation of α-synuclein in the neurons [3–8]. Point mutations on the α-synuclein gene have been identified in familial forms of PD [9, 10], and duplication or triplication of α-synuclein gene is related to PD pathogenesis [11]. Also, several post-translational modifications on α-synuclein including protein phosphorylation occur in the PD brains [12, 13]. These findings suggest that α-synuclein modification or phosphorylation (p-α-synuclein) plays a cardinal role in pathogenesis of PD.

Current treatments for PD include dopaminergic therapies aimed at managing motor symptoms. These drugs mainly provide a symptomatic attenuation without modifying the underlying pathogenic process of the disease [14–18]. Hence, it is crucial to develop more extensive and fundamental therapeutic approaches, and much research has been focused on developing disease-modifying interventions [17].

*Boswellia serrata* is a medium- to large-sized tree and is native to mountainous regions of Northern Africa, the Middle East, and India [19]. People in different parts of the world have used Boswellia resin for many centuries to cure various diseases [20, 21]. Various in vivo and in vitro studies have shown the anti-inflammatory properties of *Boswellia serrata* resin for the treatment of Crohn’s disease, ulcerative colitis, and ileitis [22–24]. In recent years, the neuroprotective effects have been reported for Boswellia-related products. It has been shown that aqueous infusions of *Boswellia serrata* significantly improve the neurodegenerative characteristics of Alzheimer’s disease in rats [25]. β-boswellic acid, the major component of *Boswellia serrata* gum, significantly increased in neurite branching, outgrowth, and tubulin polymerization dynamics [26]. In addition, antioxidant activity of *Boswellia serrata* has been shown in the cerebrovascular system [27].

Some studies have demonstrated the neuroprotective effects of *Boswellia serrata* [28, 29], but its specific molecular mechanisms for the neuroprotection remain to be obscure. In this study, we investigated the neuroprotective activity of *Boswellia serrata* gum against the rotenone-induced neurotoxicity and showed AMPK activation by the extract might be associated with its pharmacological activity.

## Material and Methods

### Extraction of *Boswellia serrata* Gum

*Boswellia serrata* gum was purchased from Helmafood Co. After certification of the herb-gum identity and quality, a voucher specimen was deposited at the herbarium of the College of Pharmacy, Yeungnam University. Boswellia gum was soaked in methanol at 23 °C and ultrasonicated for 2 h to fortify the extraction. The resultant methanol solution was collected, and the extraction was repeated 5 times with new methanol. The solution was filtered and evaporated using a rotary evaporator under vacuum and stored at − 20 °C until use.

### Animals

Eight-week-old male C57BL/6 J mice (Japan SLC Inc.) were housed in microisolator cages on a 12-h light/dark cycle with free access to food and water. All of the experimental procedures were performed according to the guidelines of the institutional animal care and use committee of Yeungnam University for the humane care and use of laboratory animals (approval number: 2017–028).

### Experimental Design and Grouping

#### Phase I: Exploring Whether Boswellia Extract Increases Neuroprotective Molecules or Not

Mice in vehicle (vehicle A) and treatment group (*n* = 7) received water (2 ml/kg/day) and the extract (500 mg/kg/day) for 2 weeks via oral route, respectively. The extract was dissolved in water and made it freshly every day. The expression levels of AMPK, mammalian target of rapamycin (mTOR), beclin1, brain-derived neurotrophic factor (BDNF), and glial cell line-derived neurotrophic factor (GDNF) in the mice brains were assessed using western blotting.

#### Phase II: Evaluation of Neuroprotective Effects of Boswellia Extract Against Rotenone Neurotoxicity

Rotenone (TCI, Japan) was dissolved in DMSO and diluted using labrafil (M2125CS, Gattefossé, France). In this regard, 4 mg/kg rotenone dissolved in 10 µl DMSO and 90 µl labrafil and in total 100ul was injected intraperitoneally, daily. The animals were randomly divided into 4 groups (*n* = 7) including vehicle A + vehicle B (DMSO and larafil) (group 1), extract + vehicle B (group 2), vehicle A + rotenone (group 3), and extract + rotenone (group 4). Animals received oral treatment of vehicle A (groups 1 and 3) or Boswellia extract (groups 2 and 4) for 1 week before rotenone injection. And then the mice were injected with rotenone (4 mg/kg/day, i.p) for the next 2 weeks, while treatment of Boswellia extract was continued.

### Behavioral Tests

#### Beam Test

Training was performed every other day before rotenone injection was started. The beam consisted of four 25-cm-long segments. Width of each segment was 3.5 cm, 2.5 cm, 1.5 cm, and 0.5 cm. The beam was inclined into 15° and the end of it was placed in the home cage. The mice were placed on the beam, and the crossing time was measured 1 week and 2 weeks after the first injection of rotenone.

#### Challenging Beam Test

The challenging beam test was performed right after the beam tests. For the test, a wire mesh with 1-cm square was affixed 1 cm above the beam. The test was performed as described previously [30]. Briefly, the video was captured while the mice crossed the mesh-covered beam, and total steps and errors for each trial were counted as the video was played in slow motion (Movie maker, Microsoft). The limb slips beyond 0.5 cm below the grid surface was considered as an error.

#### Cylinder Test

The cylinder test is to examine the voluntary movement in the cylinder. To conduct the test, we placed the mouse in a clear and transparent cylinder with a diameter of 15 cm, and the number of rearings was counted for 3 min. Rearing was defined by the placement of the whole palm of forelimb on the wall of the cylinder to support body. We counted only when the mouse raised its forelimbs above shoulder level and removed both of the forelimbs from the cylinder before another rearing.

### Sampling

The mice were euthanized by CO_2_ asphyxiation after the regimens or behavioral tests were completed in the phases I and II. The left hemisphere of each brain was fixed in 4% paraformaldehyde solution for the immunohistochemical staining, and the right hemisphere was used for analyses of neurochemicals and proteins.

### Western Blots

Samples were lysed in ice-cold RIPA buffer (Thermo Fisher Scientific Inc., USA) containing 1% protease inhibitor cocktail (Thermo Fisher Scientific Inc.), 1% PMSF, and 8% Triton X-100 (10%) using sonicator. The tissue homogenate was centrifuged at 4 °C for 20 min, and the supernatant was transferred to a fresh tube. To determine the protein concentration, the BCA protein assay kit was used (Thermo Fisher Scientific Inc.). An equal amount of protein (30 µg) was mixed with a loading buffer (0.125 M Tris–HCl, pH 6.8, 20% glycerol, 4% SDS, 10% mercaptoethanol, and 0.002% bromophenol blue) and boiled for 5 min. Proteins were electrophoresed on 12% SDS–polyacrylamide gel and transferred to polyvinylidene difluoride membranes (PVDF) (Millipore Corporation, Temecula, CA). The membrane was blocked using 5% skim milk in tris-buffered saline (0.1% Tween 20, TBST) for 1 h. To detect phosphoproteins, the membrane was blocked in 5% bovine serum albumin (BSA) in TBST. Next, the membrane was incubated overnight at 4 °C with specific primary antibodies against pan AMPK and p-AMPK (1:2000, Cell Signaling Technology Inc., #2532 and #2535), pan mTOR, phosphorylated mTOR (1:1000, Cell Signaling Technology, Inc., #2972 and #2971), GDNF (1;1000, Abcam, Cambridge: #ab18956), BDNF (1:1000, Abcam, Cambridge, #ab108319), phosphorylated α-synuclein (1:2000, Santa Cruz Biotechnology Inc., #sc-135638), α-synuclein (1:2000: BD Biosciences, #610,787), and beclin 1 (1:1000, Santa Cruz Biotechnology Inc, #sc-48381). β-actin was considered as housekeeping protein. After washed three times with TBST, the membrane was incubated with a secondary antibody for 1 h at 23 °C. Following rinse in TBST, the membrane was treated with enhanced chemiluminescence reagents (Thermo Fisher Scientific) and exposed in a luminescence image analyzer (Fusion Solo, Vilber Lourmat, France) to detect the signal for the immunoreactive complex. Finally, the density of blots was analyzed using ImageJ software (NIH, USA).

### Immunohistochemistry

After fix in 4% paraformaldehyde for 24 h, the brains were transferred into 30% sucrose in phosphate-buffered saline (PBS) at 4 °C. The coronal brain Sects. (30 µm) were made on a freezing sliding microtome (Microm HM 450, Thermo Scientific) and were collected in cryoprotectant solution. Next, immunostaining was performed with free-floating brain sections. At first, endogenous peroxidase of the brain section was quenched by incubation in 3% hydrogen peroxide for 20 min. Then, the sections were incubated at 4 °C for 24 h with the primary antibodies against tyrosine hydroxylase (TH), (1:1000, Thermo Fisher Scientific, # PA1-4679, goat), rabbit anti-ionized calcium binding adaptor molecule 1 (Iba-1) (1:3000 Wako Pure Chemical Industries, # 019–19,741), rabbit anti-phosphorylated α-synuclein (1;1000, Santa Cruz Biotechnology Inc., # sc-135638), or α-synuclein (1:3000, BD Biosciences, # 610,787) in KPBS containing 0.4% triton X-100. After rinse in KPBS, the sections were treated with a biotinylated secondary antibody (1:1000, Vector Laboratories Inc., Burlingame, USA) diluted in KPBS containing 0.4% Triton X-100 for 2 h. Following washes in KPBS, sections were immersed in avidin–biotin-peroxidase complex (ABC) (10ul of solution A + 10ul solution B in 10 ml of KPBS) (Vector Laboratories, Inc.) at 23˚C for 1 h. The immunocomplex was visualized using a diaminobenzidine (Sigma-Aldrich) solution (4 mg/10 ml KPBS + 5 µl of 30% H_2_O_2_). Subsequently, the immunostained sections were mounted onto gelatin-coated slides, dehydrated in ethanol, and cleared in xylene and coverslipped before observing under a light microscope.

Finally, the number of TH positive cells and hematoxylin-stained cells were determined blindly using stereological counting with every 6 sections covering the whole substantia nigra (*n* = 7/group) (BX41 TF, Olympus, Tokyo, Japan). The optical density (OD) of the striatal dopaminergic fibers and phosphorylated α-synuclein were analyzed using ImageJ-2 software [31]. Briefly, we picked every 6th section of the SN or every 12th section of the striatum covering rostral to caudal parts of the brain regions, and immunohistochemical stainings were performed as aforementioned. After taken microscopical photos, the color of the objects was converted into gray scales and the density was measured using the software.

The number of activated microglia was counted blindly by using ImageJ-2 software [30]. Briefly, brain sections containing the SN following adjustment of threshold value to 200, and the number of pixels above the threshold was measured automatically.

### Neurochemical Analysis

HPLC (1260 Infinity, Agilent technologies) was used to measure striatal levels of DA and its metabolites as described previously [30, 32, 33]. Briefly, the stratum was carefully weighed, prior to homogenization in 100 µl of 0.1 M perchloric acid. The homogenate was centrifuged for 20 min at 4 °C, and the supernatant was transferred to a fresh tube. At the same time, stock standard solution for DA, 3,4-dihydroxyphenylacetic acid (DOPAC), homovanillic acid (HVA), and serotonin (5-HT) were prepared in 0.1 M perchloric acid, and series of standards solution were freshly prepared by diluting the stock solution in the mobile phase. The sample or standard solution was injected into the mobile phase (75-mM sodium phosphate monobasic, 1.7-mM 1-octanesulfonic acid, 100-µl/L triethylamine, 25-µM EDTA, 15% acetonitrile) with the flow rate of 0.6 ml/min. DA, DOPAC, HVA, and 5-HT were detected by the electrochemical detector (Coulochem III, Thermo Scientific). Finally, the results of the analyses were normalized to weight of the wet brain tissue.

### Cell Culture and Treatment

SH-SY5Y neuroblastoma cells were cultured in Dulbecco’s modified Eagle’s medium (DMEM) (Invitrogen, Carlsbad, CA) containing 10% FBS (GIBCO Laboratories, Grand Island, NY) and 100 U/ml penicillin, 100 mg/l streptomycin at 37 °C, in 5%, CO_2_ humidified incubator.

In order to investigate effects β-boswellic acid (Sigma-Aldrich, #80,342) against rotenone-induced cell death, SH-SY5Y cells were seeded in 96-well plates at a density of 1 × 10^4^ cells per well. Twenty-four hours later, the cells were treated with different concentrations of β-Boswellic acid (from 1 to 1000 nM) for 3 h before rotenone exposure. Then, the cells treated with 10-µM rotenone for 12 h. Cell viability was determined by the addition of cell counting kit 8 (CCK-8, Dojindo, #CK04-05) for 30 min. The absorption was read at 490 nm using a microplate reader (Multiskan GO, Thermo Scientific). The number of viable cells was expressed as percentage of the control. We selected 100 nM of β-Boswellic acid for the next experiment, since maximal neuroprotection was exhibited at this concentration.

In order to investigate effects of β-Boswellic acid in expression of p-α-synuclein and p-AMPK, SH-SY5Y cells were plated with 4 × 10^5^ cell density in a 35-mm dish and incubated for 24 h. Then, cells were treated 100 nM with β-Boswellic acid for 3 h and followed by the treatment with 10-µM rotenone for 12 h. Cell lysates were collected with ice cold RIPA buffer (Thermo Fisher Scientific Inc., USA) containing 1% protease inhibitor cocktail (Thermo Fisher Scientific Inc., USA), 1% PMSF, and 8% Triton X-100 (10%), then incubated on ice for 15 min and stored at − 20 °C overnight. Samples were centrifuged at 13,000 rpm at 4 °C for 30 min to obtain the SDS-soluble fraction. Protein concentrations of cell lysates were determined using the BCA protein assay (Thermo Scientific) by comparison with BSA standards. Protein samples (35 µg) were electrophoresed through 12% SDS–polyacrylamide gels and transferred to PVDF membranes (Millipore Corporation, Temecula, CA). The membranes were blocked with 5% skim-milk in tris-buffered saline (pH 8.0) for 30 min, then incubated with the appropriate primary antibodies at 4 °C overnight; anti-p-α-synuclein (1:2000, Santa Cruz Biotechnology Inc., #sc-135638), anti-α-synuclein (1:2000, BD Biosciences, #610,787), anti-pAMPK (1:2000, Cell Signaling Technology Inc., #2535), anti-AMPK (1:2000, Cell Signaling Technology Inc., #2532), or anti-actin (1:5000, Cytoskeleton #AAN01). The membranes were incubated for 1 h at room temperature with secondary antibodies (1:5000, HRP-conjugated goat anti-rabbit, Merck Milipore, AP130), with enhanced chemiluminescence reagents (Thermo Fisher Scientific), and exposed in a luminescence image analyzer (Fusion Solo, Vilber Lourmat, France) to detect the signal for the immunoreactive complex. Quantitation of blots was performed by densitometry using ImageJ (NIH).

### Statistical Analysis

All the data in the present study were expressed as the mean ± SEM. The statistical analysis was performed using Student’s *t* test or one-way ANOVA followed by the Student–Newman–Keuls comparison method to calculate differences between groups (GraphPad Prism 4.0, San Diego, CA). *P* values less than 0.05 were considered statistically significant.

## Results

### AMPK Activation and its Effects on Downstream Signaling Pathway by Boswellia Extract

**Fig. 1:**
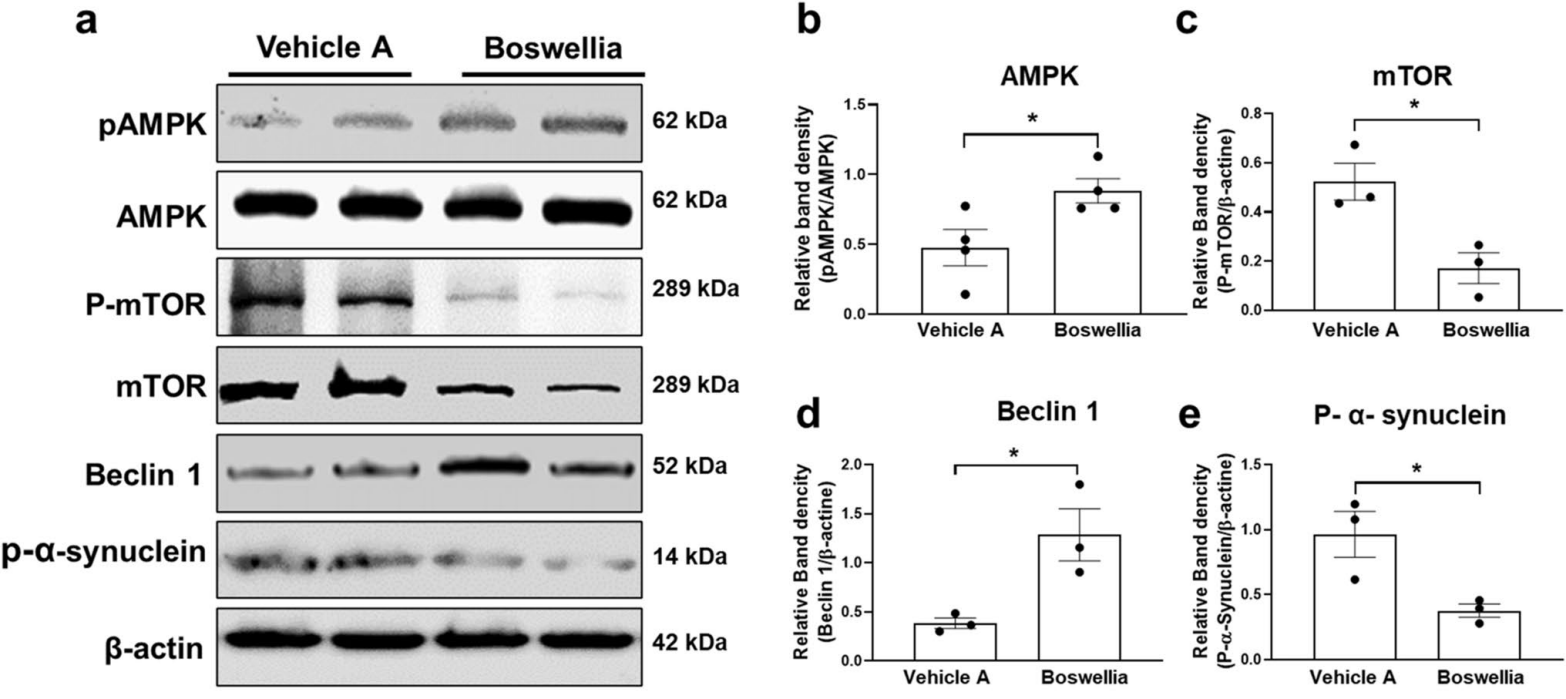
Boswellia extract activates AMPK and its downstream pathways in the striatum. C57BL/6 J mice were administered either with vehicle A or B swellia extract (500 mg/kg/day) for 2 weeks. **a** Western blots for AMPK, pAMPK, mTOR, phospho-mTOR, beclin 1, and p-α-synuclein (as a loading control) were performed with the striatal lysates following the treatment. Quantification of the blots for **b** AMPK, **c** mTOR, **d** beclin1, and **e** p-α-synuclein was conducted using Image J (NIH). *P < 0.05 All values represent mean ± SEM (*n* = 3)

It has been observed that the AMPK activation is related to the dopaminergic neuroprotection and stimulation of autophagy activity and elevation of neurotrophic factor expression [32]. Thus, we attempted to analyze effects of Boswellia gum extract on the AMPK pathway. Boswellia gum extract was administered to C57BL/6 J mice for 2 weeks, and then, western blot was performed with the striatum tissue. The results revealed that the extract significantly increased phosphorylation of AMPK in the striatum (Fig. 1a, b; *p* < *0.05*). In contrast, there was a downregulation of phosphorylated mammalian target of rapamycin (p-mTOR) and total mTOR (Fig. 1a, c; *p* < *0.05*). Moreover, Boswellia treatment increased the levels of beclin1, a marker for autophagy significantly in the striatal tissue (Fig. 1a, e; *p* < *0.05*). Moreover, Boswellia treatment decreased phosphorylation of α-synuclein in the striatum (Fig. 1a, e; *p* < *0.05*).

**Fig. 2:**
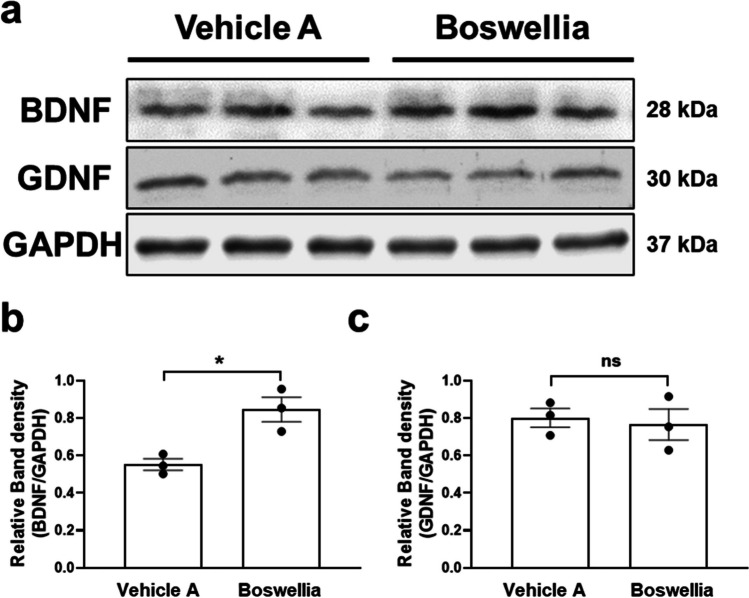
Boswellia extract upregulates the striatal level of BDNF. C57BL/6 J mice were administered either with vehicle or Boswellia extract (500 mg/kg/day) for 2 weeks. **a** Western blots for BDNF and GDNF were performed with the striatal lysates following the treatment. Quantification of the level of **b** BDNF and **c** GDNF was conducted using ImageJ software (NIH). *P < 0.05. All values represent mean ± SEM

Next, we performed western blot for BDNF and GDNF which are neurotrophic factors related to neuronal survival and neuronal differentiation. The results demonstrated that Boswellia gum extract upregulated the expression of BDNF in the striatum (Fig. 2a, b; *p* < *0.05*), while GDNF level was not significantly changed after the extract treatment (Fig. 2a, c; *P* = 0.7315).

### The Dopaminergic Neuroprotective Effect of Boswellia Extract in the Rotenone-Administered Brain

**Fig. 3:**
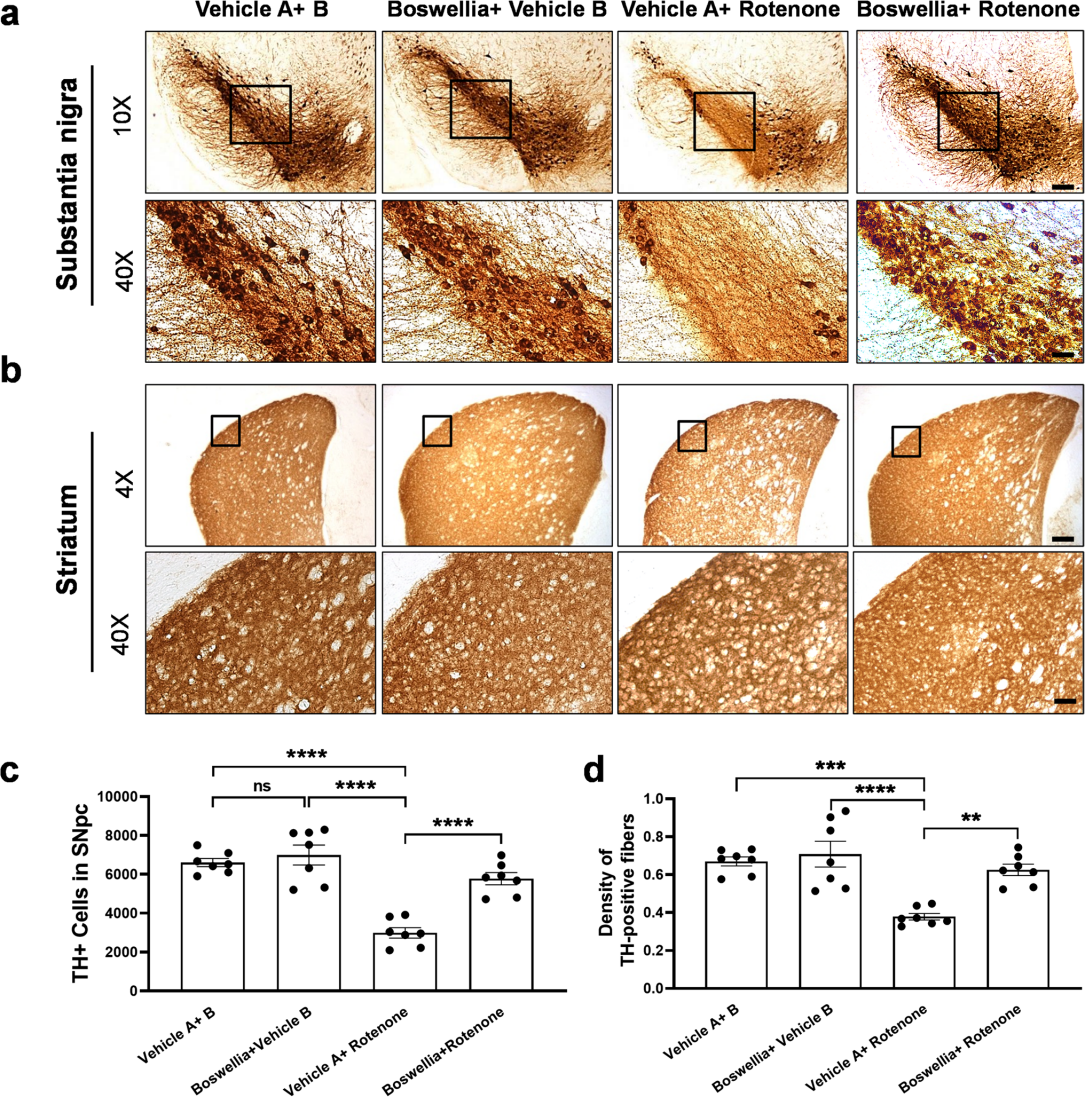
Boswellia extract attenuates rotenone-induced dopaminergic neurodegeneration. Animals received treatment with Boswellia extract (500 mg/kg/day/oral) for 2 weeks and then injection of rotenone (4 mg/kg/day, i.p) along with treatment of Boswellia extract for additional 2 weeks. Immunostainings for TH were performed to visualize the dopaminergic neurons in the substantia nigra and the TH-positive fibers in the striatum. **a** The results show Boswellia extract spares the dopaminergic neurons from rotenone-caused neuronal loss and **b** deletion of TH-positive fibers. **c** Stereological counts of TH-positive neurons in the substantia nigra. **d** Quantification of TH-positive fiber density in the striatum. ns = *P* > 0.05, **P ≤ 0.01, ***P ≤ 0.001, and ****P ≤ 0.0001. All values represent mean ± SEM (*n* = 7). Scale bars: 150 μm (4 ×) and 25 μm (20 ×)

In order to assess the protective effects of Boswellia extract on the dopaminergic neurons, we performed immunohistochemical staining for TH with the brain sections of substantia nigra and striatum. We observed abundant TH-positive neurons in the substantia nigra and TH-positive fibers in the striatum of control (Fig. 3a, c). Boswellia extract itself did not significantly affect the population of the dopaminergic neurons in the nigrostriatal pathway. In contrast, rotenone injection caused a significance loss of the nigral dopaminergic neurons (Fig. 3a, c; *P* < 0.0001) and striatal TH + fibers as compared with control (Fig. 3b, d; *P* < 0.0001). Importantly, treatment with Boswellia extract significantly attenuated the rotenone-mediated deletion of the dopaminergic neurons (Fig. 3a, c; *P* < 0.0001) and of the striatal TH + fibers (Fig. 3b, d; *P* < 0.01).

### Effects of Extract on Monoamine Levels After Rotenone-Induced Neurotoxicity

**Fig. 4:**
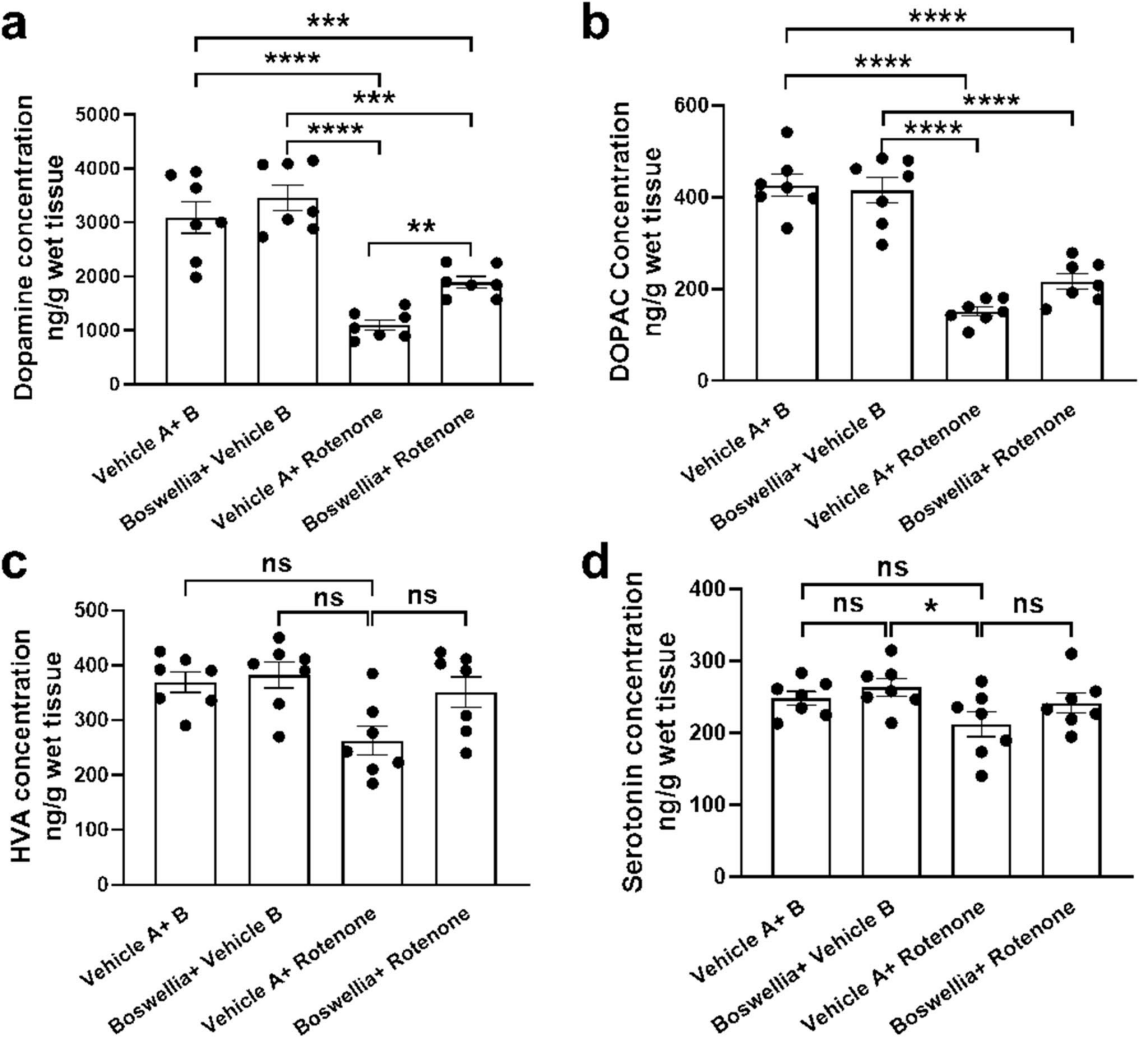
Boswellia extract mitigates rotenone-induced decrease in dopamine and its metabolite. HPLC analyses were carried out with the striatum tissues to assess levels of DA, DOPAC, HVA, and 5-HT. Rotenone markedly decreased **a** DA and **b** DOPAC, but not **c** HVA and **d** 5-HT. Boswellia ameliorated the rotenone-induced reduction of striatal levels of DA and DOPAC. ns = *P* > 0.05, **P ≤ 0.01, ***P ≤ 0.001, and ****P ≤ 0.0001. All values represent mean ± SEM (*n* = 7)

Neurochemical analysis using HPLC was conducted to measure levels of monoamine neurochemicals in the striatum. Boswellia extract itself did not cause any alterations in the neurochemical concentration in the striatum (Fig. 4a–c; *P* > 0.05). In contrast, rotenone injection for 2 weeks caused extensive loss of DA (~ 60%) (Fig. 4a; *P* < 0.0001) and DOPAC (~ 65%) (Fig. 4b; *P* < 0.0001). In addition, there was a trend toward decrease in HVA level after rotenone challenge; however, it was not statistically significant (Fig. 4c, *P* = 0.8318). Level of 5-HT in the striatum was not significantly affected by rotenone (Fig. 4d; *P* = 0.7808). Importantly, Boswellia treatment attenuated rotenone-induced depletion of DA and DOPAC (Fig. 4a, b; *P* < 0.01).

### Effects of Boswellia Extract on Behavioral Impairment Caused by Rotenone

**Fig. 5:**
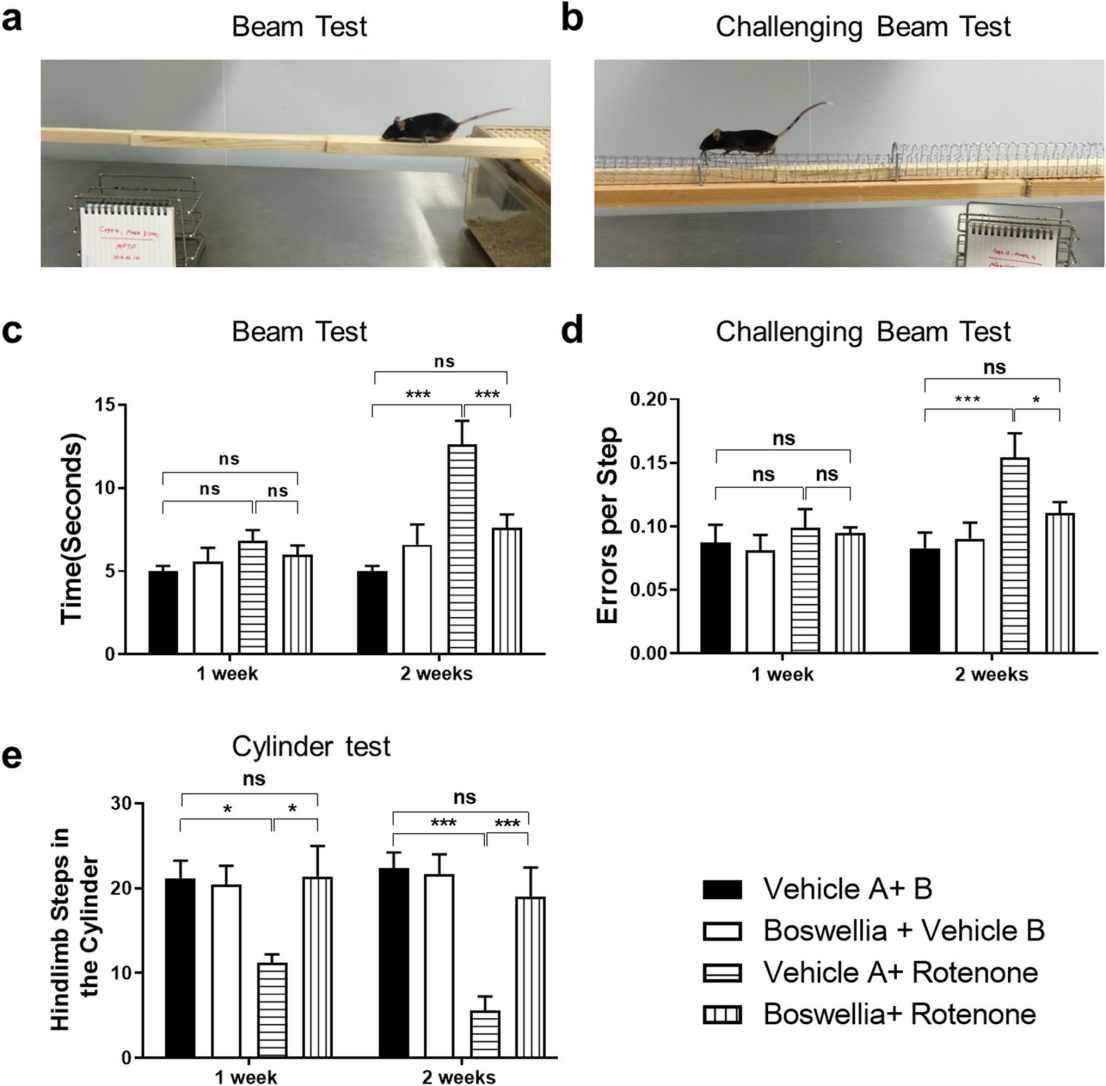
Amelioration of rotenone-induced motor deficits by Boswellia extract. To evaluate motor function of the mice after rotenone injection, behavioral tests were performed. **a**, **c** For the beam walking test, the time to reach home cage was recorded. **b**, **d** For the challenging beam test, the number of total slips of the mice was monitored. **e** The number of rearings of mice within 3 min at 7 days and 14 days after rotenone injection was counted. *P < 0.05, ***P < 0.001. All values represent mean ± SEM (*n* = 7)

We performed three different kinds of behavioral tests including the beam test, challenging beam test, and cylinder test to assess the motor function of the animals. Results of the beam test showed that there was no significant change in the performance of the animals at week 1 after rotenone injection (Fig. 5; *P* > 0.05). However, rotenone injection significantly increased the time to cross the beam at 2 weeks (Fig. 5a, c; *P* < 0.001), which was mitigated by the Boswellia extract treatment (Fig. 5a, c; *P* < 0.001). Similarly, the challenging beam test displayed that the number of rotenone-induced errors in stepping was significantly increased at week 2 of rotenone injection (Fig. 5b, d; *P* < 0.001). The motor dysfunction was attenuated by the treatment of Boswellia extract (Fig. 5b, d; *P* < 0.05). The cylinder test revealed that the number of rearings within 3 min decreased significantly at both week 1 (Fig. 5e; *P* < 0.05) and 2 after rotenone injection (Fig. 5e; *P* < 0.001). The motor deficit was completely prevented by the treatment of Boswellia extract (Fig. 5e; *P* < 0.001).

### Effects of Boswellia Extract on Microglial Activation Mediated by Rotenone

**Fig. 6:**
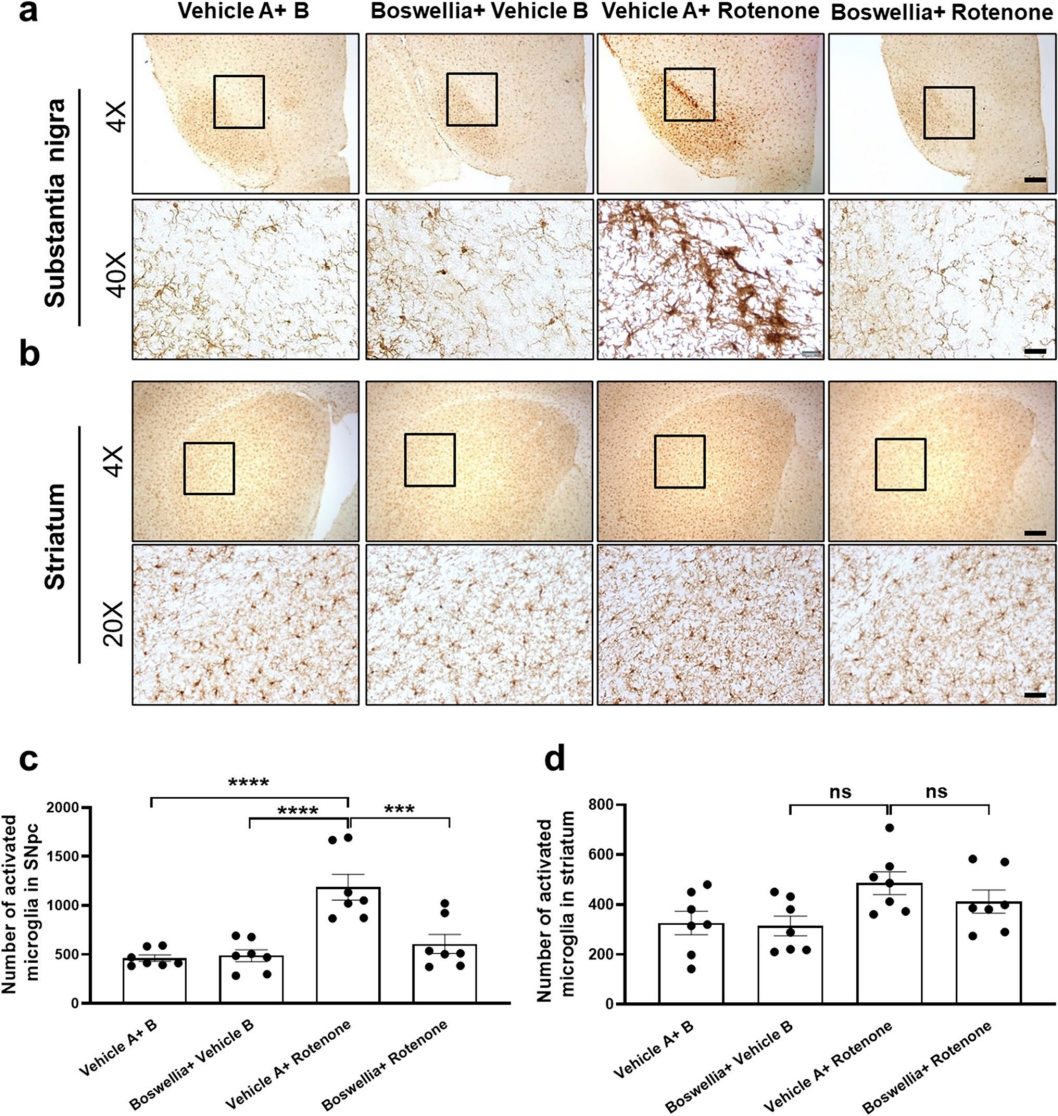
Boswellia extract attenuates rotenone-induced microglial activation. At 14 days after rotenone administration, mice were euthanized and immunohistochemical analysis for Iba-1, a marker for microglia, were performed to examine change of their phenotype. Immunohistochemical staining showed that clear rotenone-induced activation of microglia in the substantia nigra (**a**) and mild activation in the striatum (**b**), which was attenuated by Boswellia extract. Quantification of density of Iba-1 stainings in the substantia nigra (**c**) and the striatum (**d**). ns = *P* > 0.05, ***P ≤ 0.001, ****P ≤ 0.0001. All values represent mean ± SEM (*n* = 7). Scale bars: 150 μm (4 ×); 50 μm (20 ×) and 12.3 μm (40 ×)

To study if rotenone induces microglial activation, and Boswellia extract can ameliorate the microglial activation, we immunostained for Iba-1, a marker for microglia using the brain sections containing the substantia nigra and striatum. Our results showed there was no marked difference between control and Boswellia-treated group (Fig. 6a–d; *P* > 0.05). After exposed to rotenone, activated microglia were evidently increased in the substantia nigra, which are represented by shorter branches and larger cell bodies compared with quiescent microglia (Fig. 6a, c; *P* < 0.0001). In contrast, the rotenone-induced microglial activation was mild in the striatum (Fig. 6b, d; *P* > 0.05). Boswellia extract significantly attenuated the microglial activation in the substantia nigra (Fig. 6a, c; *P* < 0.001).

### Effects of Boswellia Extract on α-Synuclein Phosphorylation Following Rotenone Treatment

**Fig. 7:**
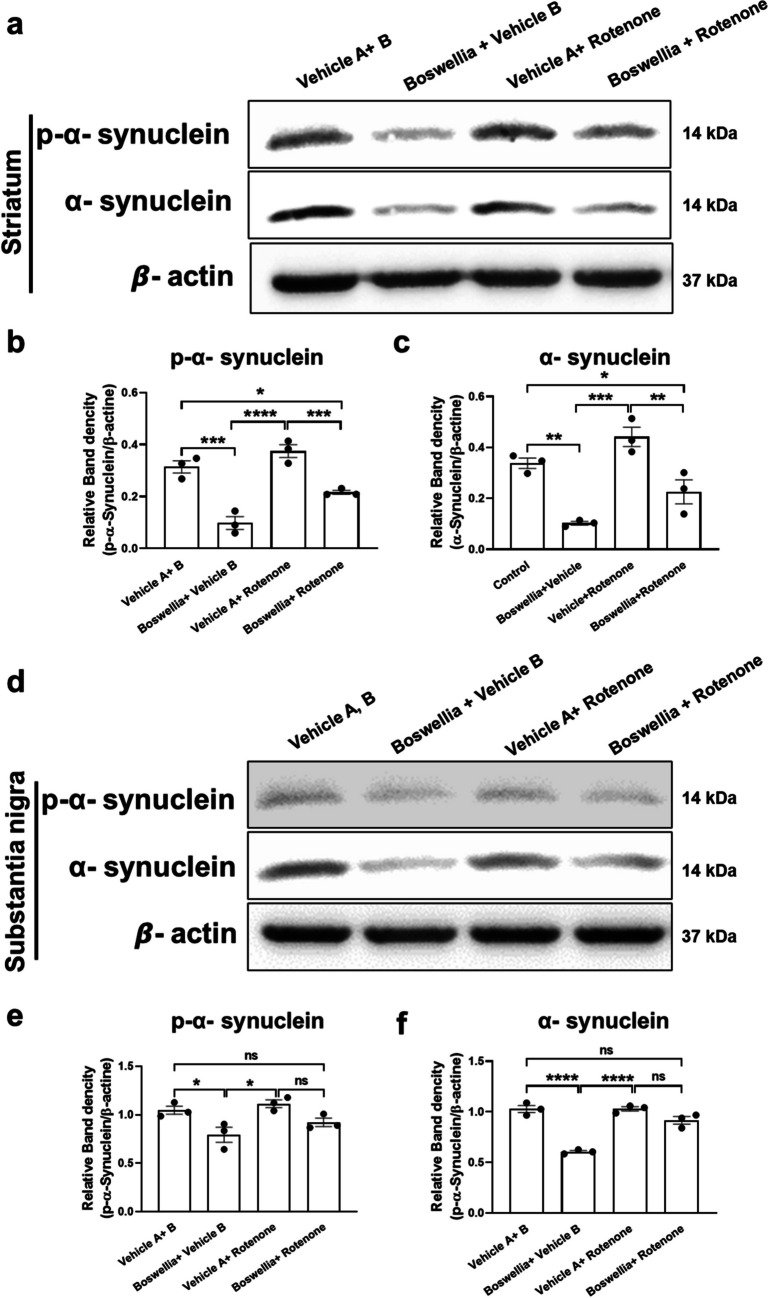
Boswellia extract decreases levels of p-α-synuclein and pan α-synuclein in the brain. The result of western blotting for p-α-synuclein and pan α-synuclein using the striatal lysates (**a**) and its quantification (**b**, **c**). The result of western blotting for phosphorylated α-synuclein and pan α-synuclein using the nigral lysates (**d**) and its quantification (**e**, **f**). *P < 0.05. All values represent mean ± SEM (*n* = 3)

We also examined whether levels of pan-α-synuclein and p-α-synuclein following Boswellia extract administration or rotenone injection. Thus, we conducted western blot analyses with the substantia nigra and striatum. The results revealed that Boswellia extract alone significantly downregulated p-α-synuclein and pan-α-synuclein comparing to control in both striatum (Fig. 7a, b, c; *P* > 0.05) and substantia nigra (Fig. 7d, e, f; *P* > 0.05). In addition, the levels of pan α-synuclein and p-α-synuclein were significantly higher in rotenone-treated group compared to the Boswellia-treated group in both striatum (Fig. 7a, c; *P* < 0.001) and substantia nigra (Fig. 7d, f; *P* > 0.05). The animals treated with Boswellia extract and rotenone had lower levels of pan α-synuclein and p-α-synuclein only in the striatum (Fig. 7a, b, c; *P* < 0.01) comparing with the control or vehicle A + rotenone group.

**Fig. 8:**
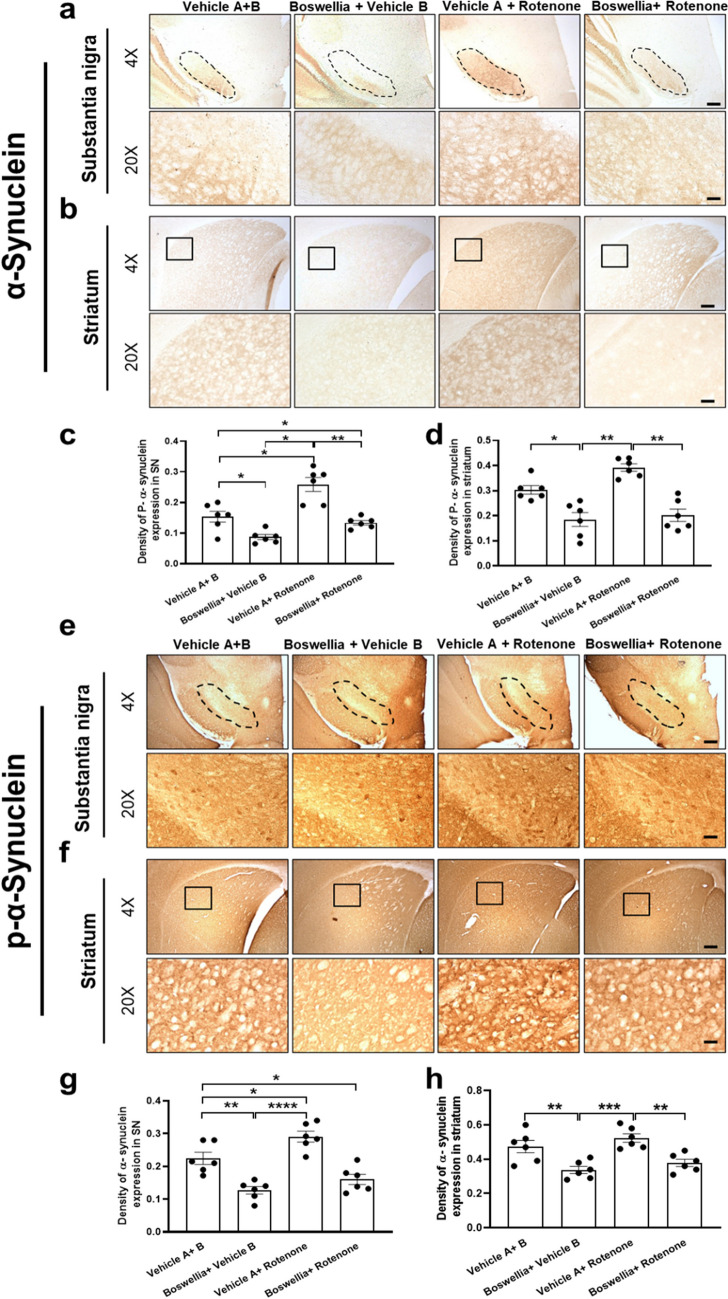
Boswellia extract decreases immunostainings for p-α-synuclein and pan α-synuclein in the brain. Effect of Boswellia extract on rotenone-induced a-synuclein phosphorylation. Immunohistochemical analysis for p-α-synuclein were performed to examine change of p-α-synuclein in **a** substantia nigra **b** striatum. **c** Quantification of p-α-synuclein expression in substantia nigra. **d** Quantification of p-α-synuclein expression in substantia striatum. Expression of α-synuclein was measured in **e** substantia nigra **f** striatum. **g** Quantification of p-α-synuclein expression in substantia nigra. **h** Quantification of p-α-synuclein expression in substantia striatum. Quantifications were conducted using ImageJ Fiji. *P < 0.05, **P < 0.01, one-way ANOVA followed by the Tukey’s multiple comparisons post hoc test. All values represent mean ± SEM (*n* = 6). × 4 scale bar 150 μm; × 20 scale bar 50 μm and × 40 scale bar 12.3 μm

To further validate the findings by western blot, we immunostained for α-synuclein to detect expression of p-α-synuclein and pan-α-synuclein in the substantia nigra and striatum. In agreement with results of the western blot, Boswellia treatment significantly decreased p-α-synuclein (Fig. 8a–d; *P* < 0.01) and pan-α-synuclein in the substantia nigra and striatum in comparison to control (Fig. 8e, f, c; *P* < 0.01). There was a mild rise in density of p-α-synuclein and pan-α-synuclein after rotenone treatment, which was completely blocked by treatment with Boswellia extract (Fig. 8a, b; *P* < 0.01). Furthermore, the number of α-synuclein aggregations went up due to the rotenone treatment in the substantia nigra and striatum (Fig. 8e–h; *P* < 0.01). However, the number these aggregations declined after Boswellia treatment in striatum (Fig. 8c, d; *P* < 0.01).

### Effects of β-Boswellic Acid in Expression of p-α-Synuclein and p-AMPK

**Fig. 9:**
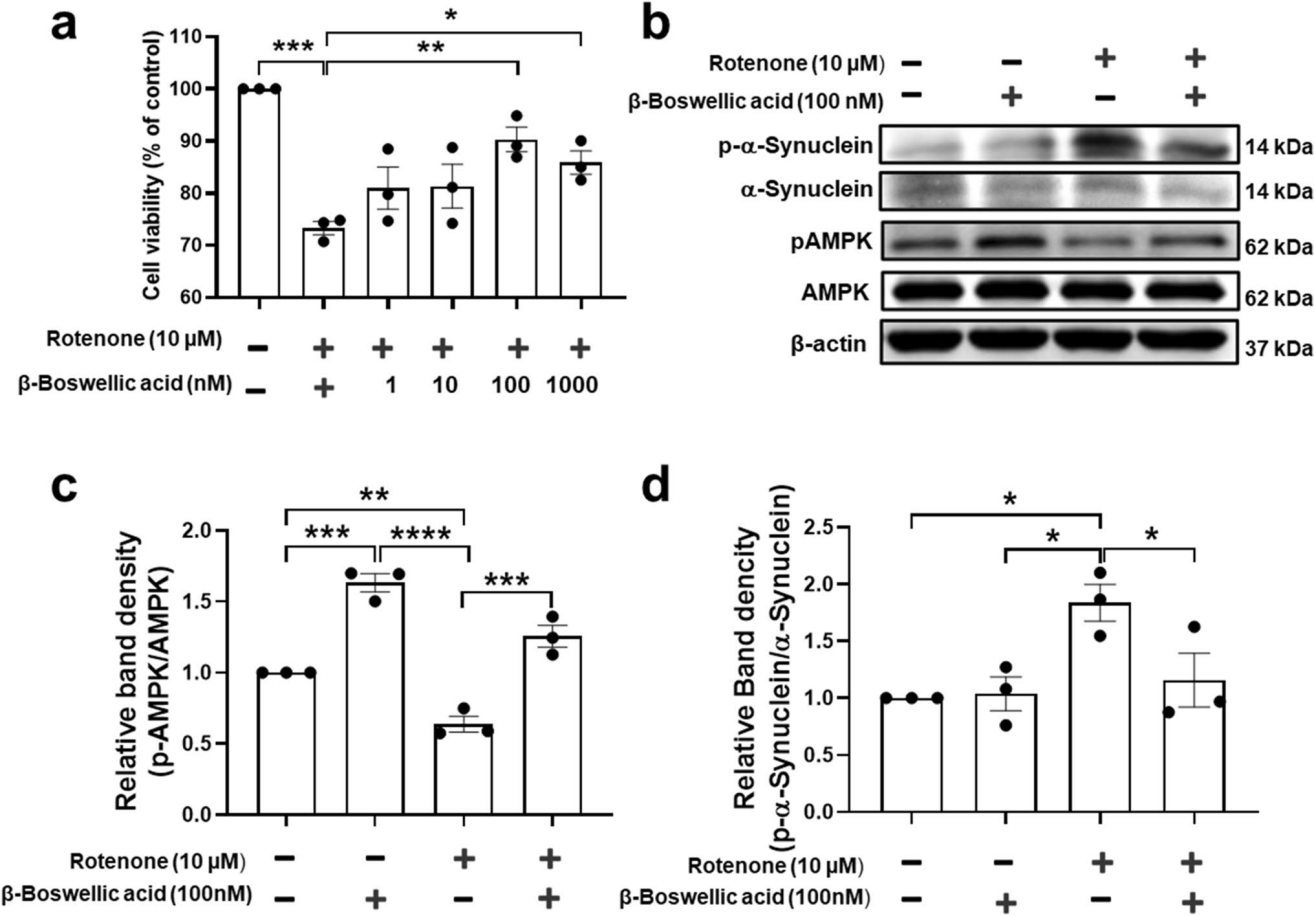
Investigating the protective properties of β-Boswellic acid in vitro. Protective properties of β-Boswellic on the against rotenone induced neuronal death was examined using CCK-8. **a** Cell toxicity assay showed that treatment with 100 nM β-Boswellic acid protects cellular death following treatment with 10-uM rotenone in SHSY5 cells. **b** Western blot analysis to investigate effects of β-Boswellic acid in p-α-synuclein and p-AMPK expression in SHSY5 cells. **c** Quantification of p-α-synuclein expression revealed that treatment with β-Boswellic acid decreased the p-α-synuclein upregulation induced by rotenone. **d** Quantification of p-AMPK expression revealed that the levels of p-AMPK increased following treatment with β-Boswellic acid. *P < 0.05, ***P < 0.001. All values represent mean ± SEM (*n* = 3)

**Fig. 10:**
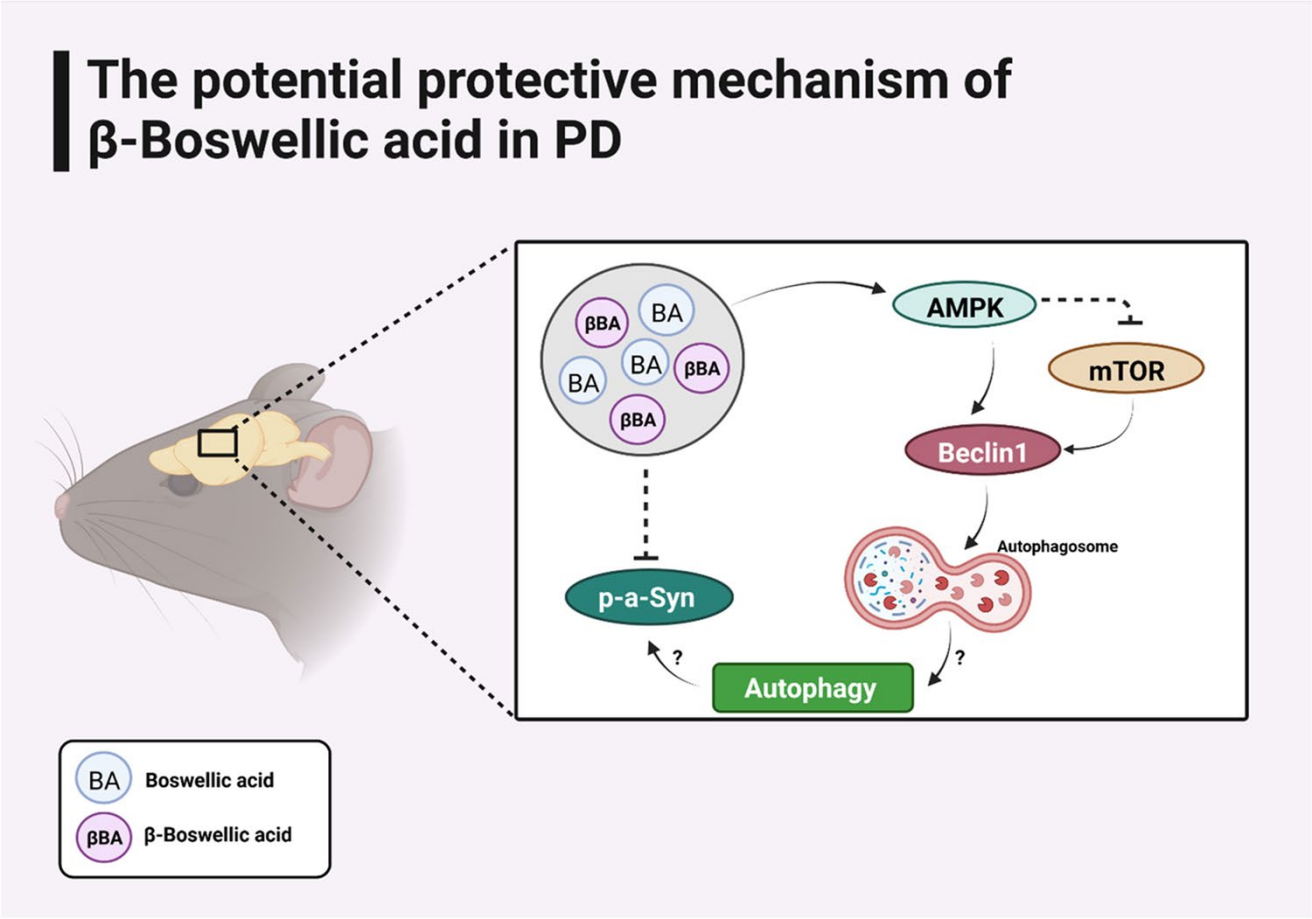
Schematic diagram illustrating the potential mechanisms underling the protective activity of *Boswellia serrata* gum (BA) and β-Boswellic acid (βBA) in PD

Even though, the primary aim of this study was to investigate the protective effects of crude Boswellia extract in PD model. However, we examined the effects of β-boswellic acid, the major component of *Boswellia serrata *gum on against rotenone-induced cells death in SH-SY5 neuroblastoma cell line. In this regard, cells were treated with β-Boswellic acid (1–1000 nM) and incubated for 3 h followed by the rotenone treatment for 12 h. CCK-8 cell viability assay demonstrated that β-boswellic acid treatment attenuated cell death induced by 10-µM rotenone in dose-dependent manner (Fig. 9a; p). Cells showed significantly higher viability following treatment with both 100 nM (Fig. 9a; *P* < 0.01) and 1000 nM (Fig. 9a; *P* < 0.05) β-Boswellic acid. Previously in this research, we showed that treatment with crude Boswellia extract resulted in increased AMPK phosphorylation (Fig. 1a, b) and decreased p-α-synuclein (Fig. 7a, f). Hear, we investigate the effects of β-Boswellic acid in expression of and p-AMPK and also α-synuclein phosphorylation in vitro in SH-SY5 cells following rotenone treatment. Western blot analyses demonstrate that cell treatment with 10-µM rotenone resulted in significant elevation in α-synuclein phosphorylation in SH-SY5 cells (Fig. 9b, d; *P* < 0.05). Treatment with 100-nM β-Boswellic acid attenuated rotenone-induced α-synuclein phosphorylation (Fig. 9b, d; *P* < 0.05), and in addition, rotenone treatment. Furthermore, treatment with β-Boswellic acid-induced AMPK phosphorylation in the SH-SY5 cells (Fig. 9b, c; *P* < 0.001). In contrast, rotenone treatment significantly decreased the phosphorylation of AMPK (Fig. 9b, c; *P* < 0.01). β-Boswellic acid attenuated rotenone-induced decreased levels of AMPK phosphorylation (Fig. 9b, c; *P* < 0.001) (Fig. 10).

## Discussion

The use of complementary and alternative medicine (CAM) has tremendously increased over the past decades and individuals worldwide trusting on them for some part of primary healthcare [34]. This and the public interest to use a traditional medicine encourages the researchers to study the potential beneficial properties of an organic plant extract are by an academic researcher. Hence, even though there are commercially available Boswelia acid, in this study, we took the holistic approach to study the potential neuroprotective benefits of consumption of Boswelia extracts derived from Boswelia serrata gum in PD. In this study, we demonstrated the neuroprotective effects of *Boswellia serrata* gum extract in vivo. Boswellia extract activated AMPK and downstream pathways which have been known to be neuroprotective. Significantly, the extract spared the nigrostriatal dopaminergic neurons from rotenone-induced dopaminergic neuronal death, as determined by immunostainings, neurochemical analysis, and behavioral tests. Further, the natural product attenuated accumulation of α-synuclein in the substantia nigra and striatum. The neuroprotective action of Boswellia extract might be attributable to activation of AMPK and its downstream signaling pathways. These findings are in agreement with those of earlier studies with Boswellia extract or Boswellic acid. Ameen et al. showed the dopaminergic neuroprotective and anti-neuroinflammatory effects of Boswellic acid in rotenone-induced parkinsonian rats [35]. Another study exhibited attenuating effects of Boswellia resin extract against MPP ± induced death of SK-N-SH cell line, the human dopaminergic neurons [29].

An intriguing finding here was that Boswellia extract significantly and evidently increased activity of AMPK as shown by increase in phosphorylation of the protein in the mice brain (Fig. 1a). This finding is in line with a previous study where they found that frankincense oil elevated phosphorylation of AMPK [36]. AMPK reciprocally suppresses activity of mTOR by phosphorylation of Raptor [37] and phosphorylation of TSC2 [38]. We also found mTOR phosphorylation was declined after treatment with Boswellia extract, further supporting our idea that Boswellia extract enhances AMPK activity.

Multiple studies demonstrated that AMPK activation was associated with the neuroprotection from various neurotoxicants. For instance, AMPK activation by metformin rescued the dopaminergic neurons from MPTP-mediated death of the nigral dopaminergic neurons [32]. Furthermore, previously it has been described that overexpression of the catalytic AMPK α subunit effectively blocked the demise of the dopaminergic neurons and behavioral impairment resulted from α-synuclein accumulation [39]. Neuron-specific ablation of AMPK gene promoted the loss of the nigral dopaminergic neurons and aberration of motor skills mediated by 6-OHDA [40]. These findings support the postulation that AMPK might be a key player in suppressing rotenone’s neurodegenerative action in this investigation.

AMPK promotes autophagy through phosphorylation of ULK1 [41]. AMPK also phosphorylates Beclin1, a multi‐domain protein that forms a complex essential for increase of autophagosome formation [42–45]. In contrast, mTOR activation suppresses autophagy activity by preventing ULK1 and disrupting interaction between ULK1 and AMPK. We herein observed AMPK activation accompanied with reduction in mTOR phosphorylation and rise in Beclin1 after treatment with Boswellia extract, suggesting elevation in autophagosome formation by the extract. In line with our results, earlier studies showed that BA145, an analog of Boswellic acid reduced mTOR phosphorylation and increased expression of Beclin1 [46].

Autophagy is one of the processes related to proteolytic degradation of the misfolded α‐synuclein [47]. Thus, pharmacological enhancement of autophagy may be a potential way to modify neurodegeneration process in PD. Indeed, autophagy inducers such as rapamycin and lithium reduced α‐synuclein accumulation and prevented neurodegeneration in vitro [48] and in vivo [49, 50]. We found reduced level of α-synuclein in the striatum of the extract-received animals, suggesting autophagy inducting by the extract might contribute to the neuroprotection against rotenone.

Another interesting finding here is that treatment with the extract enhanced expression of BDNF in the animal’s brain. BDNF is a neurotrophic factor that promotes the survival and differentiation of the mescencephalic dopaminergic neurons [51, 52]. Importantly, postmortem studies with PD brains uncovered that BDNF level was significantly reduced within the substantia nigra indicating deficiency in BDNF is related to PD pathogenesis [53]. Furthermore, BDNF could attenuate cognitive decline in Alzheimer’s disease models [54] and the dopaminergic neurodegeneration in PD models [55]. We have reported that BDNF induction by antidepressants or metformin was related to the dopaminergic neuroprotection against MPTP [30, 32] and exhibited AMPK activation by metformin led to BDNF upregulation [56]. In parallel with our studies, Khalaj-Kondori et al. showed treatment with aqueous extract of Boswellia increased the hippocampal expression of BDNF in rats [57]. These observations suggest that BDNF induction at least in part might contribute to the neuroprotective effects of Boswellia extract. These also highlight the pleiotropic actions of AMPK in the neuroprotection, as we found that BDNF upregulation depended on AMPK activation.

Previous studies demonstrated that rotenone treatment elevated numbers of α-synuclein cytoplasmatic inclusions in neurons and α-synuclein phosphorylation [58–60]. A more direct link between apoptosis and DNA damage has been described in relation to α-synuclein. Overexpression of mutants α-synuclein including α-synuclein^Ala30Pro^ or α-synuclein^Ala53Thr^ triggers apoptosis in human neuroblastoma SH-SY5Y cells [61]. Recently, a study shown increased DNA damage in two PD mouse models based on α-synuclein which was due to dopaminergic cell death by an undefined mechanism in the SNpc [62]. In the current study using immunohistochemistry, we showed that rotenone treatment increased the levels of both α-synuclein and pan α-synuclein in striatum and SN. However, the levels of α-synuclein and pan α-synuclein did not show a significant change in western blot analysis in mice brain tissues. To further study this phenomenon, we investigate the effects of rotenone on α-synuclein phosphorylation. This result was consistent with the previous findings and showed that rotenone treatment is able to increase α-synuclein phosphorylation in neurons. Animal strains and rotenone-treatment regimen might be the potential reasons for the variability of rotenone-induced synuclein pathology. Overall, the current study demonstrated that the methanolic extract of *Boswellia serrata* gum could alleviate the rotenone-induced dopaminergic neuronal loss, possibly by activation of AMPK, modulation of autophagy, and reduction of α‐synuclein accumulation. Although therapeutic potential of the extract to intervene PD neurodegeneration, further study is warranted for identification of more specific underlying mechanisms and molecular components of the natural product for the neuroprotective action. In addition, even though changes in AMPK and mTOR activity, all could affect the change of autophogosome but still autophagy is not investigated and needs further confirmation.

## Data Availability

The data sets generated during and/or analyzed during the current study are available from the corresponding author on reasonable request.
