# Correction: Methanolic Extract of Boswellia serrata Gum Protects the Nigral Dopaminergic Neurons from Rotenone-Induced Neurotoxicity

**DOI:** 10.1007/s12035-024-04481-1

**Published:** 2024-09-17

**Authors:** Sina Shadfar, Shristi Khanal, Ganesh Bohara, Geumjin Kim, Saeed Sadigh-Eteghad, Saeid Ghavami, Hyukjae Choi, Dong-Young Choi

**Affiliations:** 1 Centre for Motor Neuron Disease Research, Macquarie Medical School, Faculty of Medicine, Health and Human Sciences, Macquarie University, Sydney, 2121 NSW Australia; 2 College of Pharmacy, Yeungnam University, 280 Daehak Avenue, Gyeongsan, Gyeongbuk 38541 Republic of Korea; 3 Neurosciences Research Center, Tabriz University of Medical Sciences, Tabriz, Iran; 4 Department of Human Anatomy and Cell Science, University of Manitoba College of Medicine, Winnipeg, MB R3E 0V9 Canada; 5 Research Institutes of Oncology and Hematology, Cancer Care Manitoba-University of Manitoba, Winnipeg, MB R3E 0V9 Canada; 6 Autophagy Research Center, Shiraz University of Medical Sciences, Shiraz, 7134845794 Iran; 7 Faculty of Medicine, Katowice School of Technology, 40-555, Katowice, Poland

**Correction: Molecular Neurobiology (2022) 59:5874-5890**

10.1007/s12035-022-02943-y

The original version of this article unfortunately contained a minor error in one of the figures (Figure 3b).

The authors sincerely apologize for this oversight. We take the integrity of academic publications very seriously and have strict protocols in place to check manuscripts before submission. Unfortunately, this error resulted from an unnoticed copy-paste mistake, which was not detectable by the naked eye, which is why it was missed during the manuscript preparation and review by all authors.

The authors herby publish the corrected Figure 3.
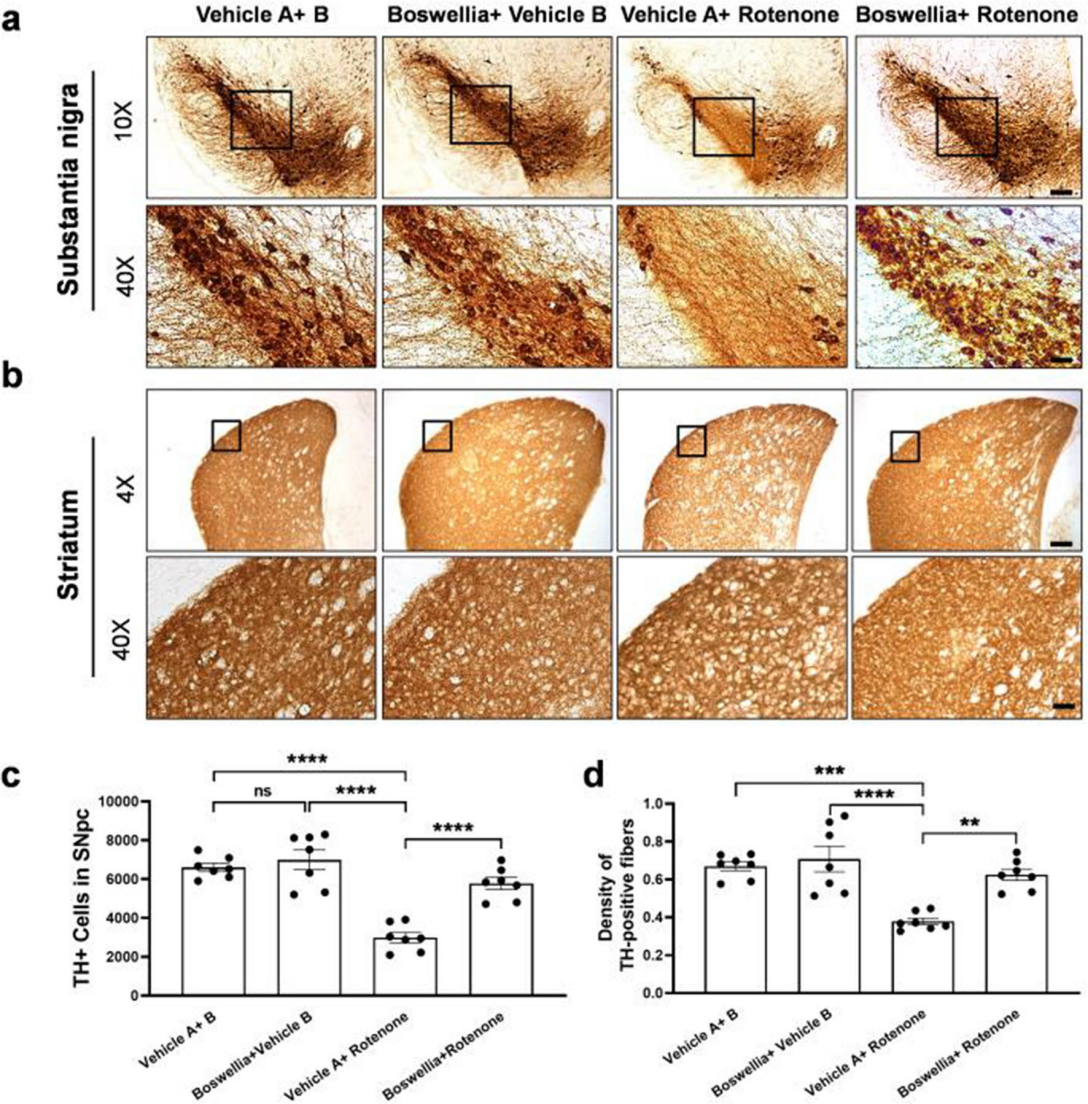


The original article has been corrected.

